# The influence of topic and systemic administration of copaiba oil on the alveolar wound healing after tooth extraction in rats

**DOI:** 10.4317/jced.51104

**Published:** 2013-10-01

**Authors:** Marco A. Dias-da-Silva, Andresa C. Pereira, Miguel CC. Marin, Miguel AC. Salgado

**Affiliations:** 1 Academic Unit of Biological Sciences, School of Dentistry, Federal University of Campina Grande (UFCG), Patos-PB, Brazil; 2 University of Vale do Paraiba (UNIVAP), São José dos Campos-SP, Brazil; 3 Department of Biosciences and Oral Diagnosis, São José dos Campos Dental School, São Paulo State University (UNESP), São José dos Campos-SP, Brazil

## Abstract

The Copaiba oil has been used as an auxiliary treatment of inflammations, skin disorders and stomach ulcers, however, in dentistry, this “alternative” medicine has not been investigated yet. The purpose of this study was to evaluate the influence of topic and systemic administration of copaiba oil on the alveolar wound healing after tooth extraction. Twenty-eight wistar male rats had their lower first molar teeth extracted. Subsequently, they were divided in four groups, according to the treatment performed: (a) alveolar socket irrigation with copaiba oil; (b) alveolar socket irrigation with physiological serum; (c) daily gavage with copaiba oil or (d) daily gavage with physiological serum. After the sacrifice, the mandibles were removed and processed in order to obtain decalcified histological sections. The results demonstrated high level of epithelial migration, small number of inflammatory cells and vascular enhancement in the animals which received systemic administration of copaiba oil. The rats treated with topic administration of copaiba oil presented ulcerations and large number of inflammatory cells. An increased bone neoformation was observed in both groups treated with copaiba oil when compared with placebo group. It could be concluded that topic or systemic administration of copaiba oil leads to a better alveolar bone healing, however the topic application on connective tissue should be carefully considered, regarding the whole socket wound healing.

** Key words:**Alveolar wound healing, oil-resin, copaiba.

## Introduction

The World Health Organization shows that over one-third of the population in developing countries lacks access to essential medicines. Consequently, the “complementary” or “alternative” medicine therapies might become a crucial tool to increase access to health care and play an important role in the therapy of many inflammatory diseases (1). The copaiba oil-resin is obtained by cutting the stem bark of Copaifera (Leguminosae), a large tree from the Amazonian region (2). It is a transparent liquid whose color varies from yellow to light brown (3).

Studies have demonstrated that copaiba oil presents anti-inflammatory (2) and antiseptic properties (4,5). This phytotherapic alternative can be used as an industrialized product or in natura, by oral administration or topic application.

Recently, Paiva et al. (6) reported that topic application of copaiba resin sped wound healing, accelerated wound contraction and increased tensile strength, facts which justify its traditional use for the treatment of wounds. However, despite copaiba oil presents desirable anti-inflammatory and antiseptic properties, there are few studies relating its use in healing process. And also, none of them evaluated hard tissues, which shows that the present study is the first to investigate the copaiba oil action in bone.

In Dentistry, studies evaluating the copaiba oil effects in different areas are extremely new. Interesting papers are seen in periodontology (7), endodontics (8,9) and dental caries (10,11). However, none investigation was found evaluating the copaiba oil resin effect in dental surgery.

Thus, the aim of this work was to evaluate the copaiba oil resin effects, by topic and systemic administration, on alveolar wound healing in rats.

## Material and Methods

The local Research Ethics Committee for Animal Use approved the protocol. Twenty-eight male wistar rats (8 weeks old) were kept in plastic cages and received tap water and food “ad libitum”. The surgical procedures, performed under intramuscular anesthesia (1ml/kg ketamine), were characterized by luxation and extraction of the first lower molars by adapted forceps. Then, six rats were submitted to 30ml topic irrigation of copaiba oil, with a syringe, once a day, during three days. Other six rats received 0.1ml of copaiba oil resin/100g of body weight, by daily gavage, with a syringe connected to a microtube conducted intraesophagically for systemic absorption, for a period of seven days. In both copaiba groups, topic (n=6) and systemic (n=6), the first dose was given immediately after surgery. Animals from the placebo group received the same doses of physiological serum during the same period. Afterward, the animals were sacrificed seven days after surgery, by cervical dislocation under anesthesia administration.

The mandibles were removed and fixed in 4% formaline solution during 48 hours. The specimens were demineralized and embedded in paraffin. Frontal semi-serial sections of mandible (6mm) were obtained and stained with hematoxylin-eosin. For the histological analysis, slices were selected from the median region of the alveolar socket.

The density of the area with newly-formed immature bone (D) was determined by point counting. A grid containing 100 equidistant points (total grid area = 2500mm2) was used in an image analyzer system (Image J; National Institutes of Health, Bethesda, USA). Analysis was performed following the dental sockets linearly with three random images, at 100× magnification. The area density corresponds to the fraction of the alveolar socket area occupied by immature bone:

D = p/3 × 2500 mm2 /100

p = the number of points hitting immature bone in three images.

Histometric data were expressed as mean and standard deviation and were submitted to two-way analysis of variance (ANOVA) and Tukey test (p<0.05).

Additional slices were stained with Sirius red, and analyzed under polarized light, in order to better understand the real condition of the connective fibers thickness and arrangement.

## Results

**Figure 1:**
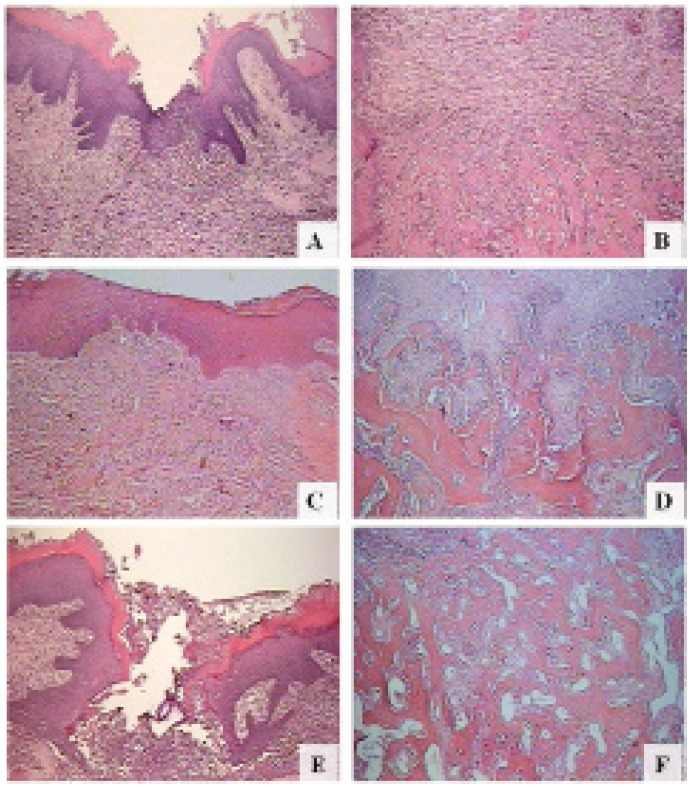
Rat alveolar socket,7 days after tooth extraction: Placebo group, epithelial migration, presence of inflammatory cells (A), and discrete immature bone remodeling (B); Systemic copaiba group, high level of epithelial migration, decreased number of inflammatory cells (C), and enhancement of immature bone remodeling, with thick bone trabeculae (D); Topic copaiba group, ulceration, large number of inflammatory cells (E), and an increased immature bone formation, represented by several bone trabeculaes (F). Hematoxylin and eosin.

Histologic analysis of alveolar sockets sections obtained from topic and systemic placebo groups presented similar healing behaviors. They were characterized by epithelial migration (Fig. 1). The group submitted to topic administration of copaiba oil demonstrated a deficient epithelial migration, with ulcerated areas and large number of inflammatory cells in the adjacent connective tissue (Fig. 1). Nevertheless, the immature bone formation was increased (Fig. 1) when compared with the samples of the placebo group (Fig. 1). The immature bone in these specimens was characterized by discrete bone formation, represented by irregularly distributed thin trabeculae (Fig. 1).

In contrast, rats treated with copaiba oil systemically showed high level of epithelial migration, small number of inflammatory cells (Fig. 1) associated to thicker bone trabeculae (Fig. 1).

Bone neoformation means and standard deviation are shown in  Table 1.

**Table 1:**
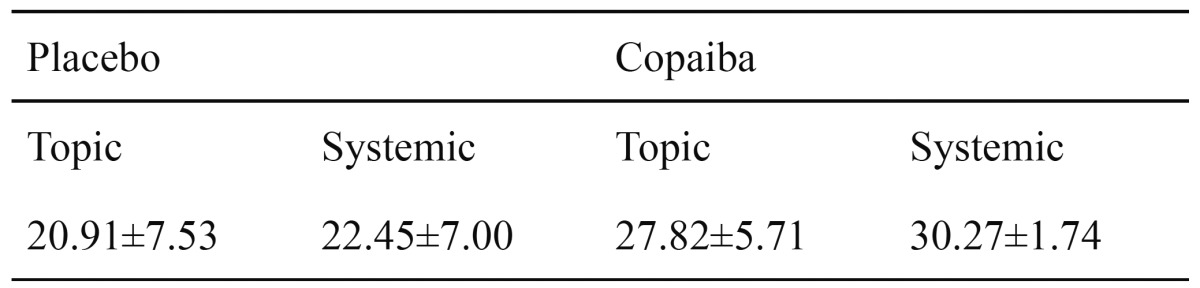
Relative frequency of bone formation from placebo and copaiba groups, topic and systemic treatment (χ ± standard deviation).

**Figure 2:**
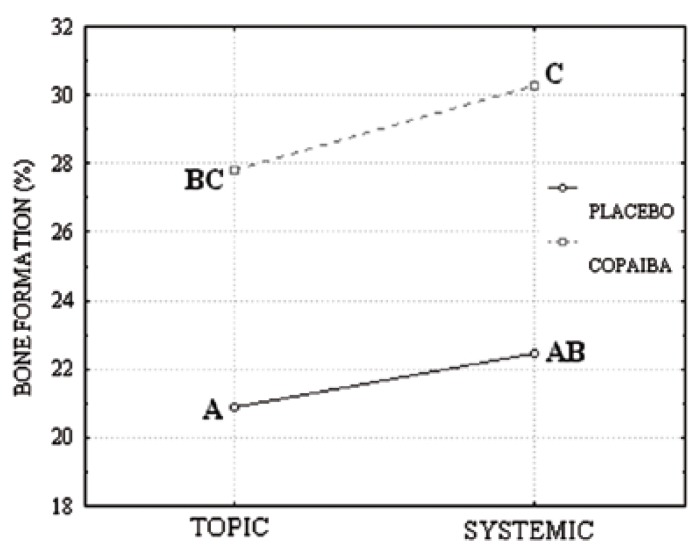
Graph for relative frequency of bone formation from placebo and copaiba groups, topic and systemic treatment.

Data were submitted to ANOVA and the interaction between all variables was not significant (p=0.80). Tukey test (p<0.05) was performed and homogeneous group formation is illustrated in fig. 2. Statistical analysis showed that the bone formation was significantly increased in both groups treated with copaiba oil when com-pared with the placebo groups. Despite the alveolar bone formation of the group systemically treated with copaiba oil is greater than the bone formation observed in the group that received topic administration of copaiba oil, they were considered statistically equivalent (Fig. 2).

**Figure 3:**
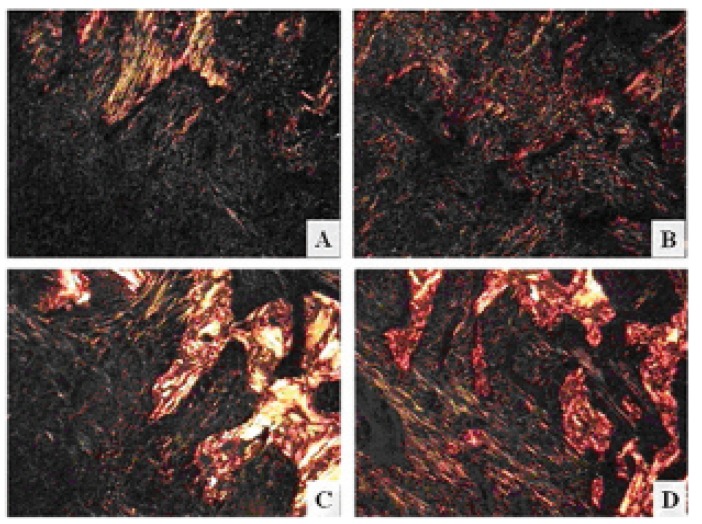
Rat alveolar socket, 7 days after tooth extraction: Polarized light images, demonstrating thicker and well organized collagen fibers, predominantly red/yellow, in systemic copaiba group (B and D) than in placebo (A and C), tending to yellow/green. Sirius Red.

These observations were confirmed after polarized light analysis, in which collagen fibers are thick and more organized in copaiba group than in placebo (Fig. 3). This same technique also allows us to confirm the better connective and osseous conditions after endogenous administration (Fig. 3).

## Discussion

The anti-inflammatory and antiseptic properties of copaiba oil were previously demonstrated by Basile et al. (12), Gomes et al. (13) and Tincusi et al. (5). Gastroprotective and wound healing effects of oleoresin (2,6) and the antinociceptive, antimicrobial, cytotoxic, and smooth muscle relaxant effects of the greater diterpenoid constituent of the kaurenoic acid were studied by several authors (14,15). Recently, Paiva et al. (1) demonstrated a protective effect of copaiba oil in the acute colitis animal model. These studies have demonstrated the protective effect of copaiba oleoresin; however, the effects of this oil on oral mucosa wound repair still remains poorly evaluated.

After tooth extraction, bone formation plays an important role in alveolar socket wound healing. The metabolism of bone tissue depends on maintaining a delicate balance between bone resorption by osteoclasts and bone formation by osteoblasts (16).

Our study was the first to demonstrate that copaiba oil-resin promotes an enhancement on alveolar socket wound healing in rats after tooth extraction. In addition, we also observed that when copaiba oil-resin was used, not only endogenous but also topic treatments lead to an increase in bone formation.

The composition of vegetal oils may vary depending on its geographic location of extraction (17). In this study we have used copaiba oil collected around Belem / PA / Brazil area. Studying the oil collected from the same area Veiga et al. (3) observed its capability to reduce edema in rats’ paw. In the same study they also demonstrated that, between several other samples collected in Brazil, the samples from this region presented the major amounts of sesquiterpenes, more specifically αbergamotene, concluding that this compound may be the responsible by copaiba oil anti-inflammatory activity.

Terpenes are known to inhibit the activity of the nuclear factor kappaB (18). The nuclear factor kappaB also known as RANK has been shown to be a key component of innate immunity (6), promoting the expression by macrophages of a set of genes involved in host defense, such as pro-inflammatory cytokines (19), NOS-2, cy-clooxygenase2, cell adhesion molecules, and various matrix metalloproteinases (20,21).

Moreover, RANK is present on the surface of osteoclast precursors and mature osteoclasts and plays a critical role in promoting osteoclast differentiation (18,21) and activation, leading to bone resorption.

Consequently, it could be suggested that the positive influence on alveolar bone formation is probably explained by the fact that copaiba oil terpenoids inhibited the RANK actions, facilitating alveolar bone formation.

It can also be considered that the understanding of wound healing process still requires further investigations to evaluate the part played by copaiba oil components in both sides of bone metabolism, by inhibition of other osteoclastogenic cytokines, as IL-1, IL-6, IL-11, IL-13, IL-17 (22) or stimulation of osteoblasts.

The present study reveals that the animals treated with topic copaiba oil presented a reduced epithelial migration and, despite copaiba oil resin antibacterial activities (15), the presence of this oil may supposedly interfere in basal membrane reconstruction or act as a lamina propria irritant agent. This data disagree with Paiva et al. (6), who reported that topic application of copaiba sped wound healing and accelerated wound contraction. Nevertheless, Paiva et al. evaluated skin wound healing, and in the present study, the topic application was inside the oral cavity. It is hypothesized that in oral cavity, the action of copaiba oil may be prejudiced by saliva presence, which could interfere on its positive role in wound healing.

Corroborating to these suppositions, our polarized light observations show that connective fibers exposed directly to the oil, by topic treatment, presented lack of organization and thickness. On the other hand, the similar regions on rats oral tissue submitted to endogenous treatment showed more mature and organized collagen fibers.

It can be concluded that the copaiba oil topic or systemic administration facilitated the maintenance of alveolar bone width and height following tooth loss, fact that is essential to the subsequent restorative procedures, such as dental implants (23). In addition, the systemic copaiba treatment seems to be more effective than the topic one, due to the best organization of the neoproduced tissue seen in Sirius red slices and the absence of ulcerations in HE slices.

Based on the present findings, it might also be inferred that the antiseptic properties of copaiba oil resin may have an important role on the results observed in the topically treated animals, moreover, these results also suggest that some copaiba oil components, such as Kaurenoic acid (the main diterpene in this oleoresin) (1), might restrain the inflammatory process and, thus, accelerate the alveolar socket repair.

The findings of this study may suggest that topic, and also, systemic administration of copaiba oil might promote better results after oral surgical interventions, due to increased bone neoformation when compared with placebo group; despite these good data, the alveolar irrigation directly on connective tissue should be carefully considered, regarding the odd oral mucosa wound healing.

## References

[1] Paiva LA, Gurgel LA, Silva RM, Tomé AR, Gramosa NV, Silveira ER (2002). Anti-inflammatory effect of kaurenoic acid, a diterpene from Copaifera langsdorffi on acetic acid-induced colitis in rats. Vascul Pharmacol.

[2] Paiva LA, Rao VS, Gramosa NV, Silveira ER (1998). Gastroprotective effect of Copaifera langsdorffii oleo-resin on experimental gastric ulcer models in rats. J Ethnopharmacol.

[3] Veiga VF, Zunino L, Calixto JB, Patitucci ML, Pinto AC (2001). Phytochemical and antioedematogenic studies of commercial copaiba oils available in Brazil. Phytother Res.

[4] Santos AO, Ueda-Nakamura T, Dias Filho BP, Veiga Junior VF, Pinto AC, Nakamura CV (2008). Antimicrobial activity of Brazilian copaiba oils obtained from different species of the Copaifera genus. Mem Inst Oswaldo Cruz.

[5] Tincusi BM, Jimenez IA, Bazzocchi IL, Moujir LM, Mamani ZA, Barroso JP (2002). Antimicrobial terpenoids from the oleoresin of the Peruvian medicinal plant Copaifera paupera. Planta Med.

[6] Paiva LA, de Alencar Cunha KM, Santos FA, Gramosa NV, Silveira ER, Rao VS (2002). Investigation on the wound healing activity of oleo-resin from Copaifera langsdorffi in rats. Phytother Res.

[7] Pereira SL, Barros CS, Salgado TD, Filho VP, Costa FN (2010). Limited benefit of copaifera oil on gingivitis progression in humans. J Contemp Dent Pract.

[8] Garrido AD, Lia RC, França SC, da Silva JF, Astolfi-Filho S, Sousa-Neto MD (2010). Laboratory evaluation of the physicochemical properties of a new root canal sealer based on Copaifera multijuga oil-resin. Int Endod J.

[9] Garcia L, Cristiane S, Wilson M, Soraya M, Lopes RA, Mônica R (2011). Biocompatibility assessment of pastes containing Copaiba oilresin, propolis, and calcium hydroxide in the subcutaneous tissue of rats. J Conserv Dent.

[10] Souza AB, Martins CH, Souza MG, Furtado NA, Heleno VC, de Sousa JP (2011). Antimicrobial activity of terpenoids from Copaifera langsdorffii Desf. against cariogenic bacteria. Phytother Res.

[11] Pieri FA, Mussi MC, Fiorini JE, Moreira MA, Schneedorf JM (2012). Bacteriostatic effect of copaiba oil (Copaifera officinalis) against Streptococcus mutans. Braz Dent J.

[12] Basile AC, Sertié JA, Freitas PC, Zanini AC (1988). Anti-inflammatory activity of oleoresin from Brazilian Copaifera. J Ethnopharmacol.

[13] Gomes NM, de Rezende CM, Fontes SP, Matheus ME, Pinto AC, Fernandes PD (2010). Characterization of the antinociceptive and anti-inflammatory activities of fractions obtained from Copaifera multijuga Hayne. J Ethnopharmacol.

[14] De Alencar Cunha KM, Paiva LA, Santos FA, Gramosa NV, Silveira ER, Rao VS (2003). Smooth muscle relaxant effect of kaurenoic acid, a diterpene from Copaifera langsdorffii on rat uterus in vitro. Phytother Res.

[15] Velikova M, Bankova V, Tsvetkova I, Kujumgiev A, Marcucci MC (2000). Antibacterial ent-kaurene from Brazilian propolis of native stingless bees. Fitoterapia.

[16] Rodan GA, Martin TJ (2000). Therapeutic approaches to bone diseases. Science.

[17] Veiga Junior VF, Rosas EC, Carvalho MV, Henriques MG, Pinto AC (2007). Chemical composition and anti-inflammatory activity of copaiba oils from Copaifera cearensis Huber ex Ducke, Copaifera reticulata Ducke and Copaifera multijuga Hayne--a comparative study. J Ethnopharmacol.

[18] Castrillo A, de Las Heras B, Hortelano S, Rodriguez B, Villar A, Bosca L (2001). Inhibition of the nuclear factor kappa B (NF-kappa B) pathway by tetracyclic kaurene diterpenes in macrophages. Specific effects on NF-kappa B-inducing kinase activity and on the coordinate activation of ERK and p38 MAPK. J Biol Chem.

[19] Ghosh S, May MJ, Kopp EB (1998). NF-kappa B and Rel proteins: evolutionarily conserved mediators of immune responses. Annu Rev Immunol.

[20] Li JJ, Westergaard C, Ghosh P, Colburn NH (1997). Inhibitors of both nuclear factor-kappaB and activator protein-1 activation block the neoplastic transformation response. Cancer Res.

[21] Mohan R, Rinehart WB, Bargagna-Mohan P, Fini ME (1998). Gelatinase B/lacZ transgenic mice, a model for mapping gelatinase B expression during developmental and injury-related tissue remodeling. J Biol Chem.

[22] Kotake S, Udagawa N, Takahashi N, Matsuzaki K, Itoh K, Ishiyama S (1999). IL-17 in synovial fluids from patients with rheumatoid arthritis is a potent stimulator of osteoclastogenesis. J Clin Invest.

[23] Wang RE, Lang NP (2012). Ridge preservation after tooth extraction. Clin Oral Implants Res.

